# “Real Sexual Harassment”: Attrition Among Police-Reported Sexual Molestation Crimes in Sweden

**DOI:** 10.1177/10778012251372551

**Published:** 2025-09-03

**Authors:** Linnea Wegerstad, Ulrika Andersson

**Affiliations:** Faculty of Law, 5193 Lund University, Lund, Sweden

**Keywords:** criminalization, preliminary investigations, sexual harassment, attrition, sexual violence

## Abstract

This article presents findings from a quantitative analysis of 215 case files regarding incidents of the crime of sexual molestation reported to the Swedish Police. We track the incidents through the criminal justice system and show a clearance rate of 9%. The clearance rate is higher when the attack is of a physical nature, the suspect is a stranger, and the incident occurs in a public place. While the statutory definition of sexual molestation is inclusive, this study shows that substantially there is a typical case of sexual harassment that is criminalized; a kind of “real sexual harassment” emerges.

## Introduction

While #MeToo highlighted the broad spectrum of violations against sexual integrity, much activism and legal research in Sweden in the last few decades has focused on rape, culminating in a rape law reform in 2018 (Wegerstad, 2021). This article focuses on other intimate intrusions of a gendered and sexual nature reported to the Swedish Police Authority that we describe under the umbrella term “sexual harassment.” Such intimate intrusions seem understudied in comparison to rape. While we will distinguish sexual harassment from rape, sexual abuse, and sexual assault for the purposes of this article, it is important to acknowledge that all such intimate intrusions are part of a continuum of women's experiences of sexual violence (Kelly, 1988). A key idea here is that sexual violence consists of a series of events that, from the viewpoint of women, cannot be readily distinguished nor hierarchically ranked according to their seriousness. There is no clear-cut moment when one form of sexual violence “turns into” another, and what they all have in common is that they are concerned with the control of women (Kelly, 1988, p. 76). The following list illustrates the kinds of behavior this article is about: “Visual forms of harassment include leering, menacing staring and sexual gestures; verbal forms include whistles, use of innuendo and gossip, sexual joking, propositioning and explicitly threatening remarks; physical forms include unwanted proximity, touching, pinching, patting, deliberately brushing close, grabbing” (Kelly, 1988, p. 103). Additional forms of sexual harassment occur in the digital realm. Both sexual harassment in public spaces and the routine, strategic decisions women make to avoid men's intrusive behavior place effective limits on women's freedom (Vera-Gray & Kelly, 2020, p. 266).

Legally, sexual harassment has traditionally been situated within the domain of workplace regulation. It has been conceptualized as a form of discrimination and has been defined in laws that regulate work (Niemi, 2019). There are some forms of intimate intrusions that are also often criminalized, but they generally remain on the periphery of criminal law. This is owing to the inherent logic of criminal law, which grades sexual violence according to perceived seriousness. Thus, rape, for example, is considered a crime of the most severe kind, while verbal insults are a crime of the least severe kind (Wegerstad, 2022). Sexual violence is often not reported to the Police Authority (Brottsförebyggande rådet [BRÅ], 2019a, p. 27). One Swedish study showed that only 8% of women who had experienced sexual violence reported it to the police (Lundgren et al., 2001, p. 50). Another study of women's experiences of sexual harassment in public spaces found that 98.5% of the women exposed to such harassment had not reported their experiences to the police (Mellgren et al., 2017). Yet, as Hansen et al. (2020) have pointed out regarding rape, reporting incidents of sexual violence is strongly encouraged by official discourse in the Nordic countries. Given this expectation, it is important to map what happens when such encouragement is taken seriously and incidents are indeed reported to the police.

While sexual harassment behavior is often not criminalized as such, laws usually exist that cover various forms of intrusive behavior (McGlynn, 2024; Wegerstad, 2022). In addition, intimate intrusions are increasingly being criminalized across Europe. Germany introduced a sexual harassment offense in 2016 (Hörnle, 2017), and Finland in 2014 (Niemi, 2019). New forms of sexual harassment driven by technology development, such as cyberflashing and image-based sexual abuse, have led both to new legal practices and to debate about whether and how to criminalize such behaviors (McGlynn, 2024). Sexual harassment in public spaces and street harassment have also garnered more attention in recent years (Fileborn, 2019; Fileborn & Vera-Gray, 2017; Stefansen, 2019; Vera-Gray, 2017). Whereas intimate intrusions in the workplace and in intimate relationships have both been rather thoroughly addressed in research and policy, public space seems to be “one of the most understudied contexts” for sexual harassment (Vera-Gray & Kelly, 2020, p. 265).

Everyday intimate intrusions are, in large part, already formally criminalized in Sweden (Wegerstad, 2022). However, we know less about how the criminal justice system responds to sexual harassment in practice. Studies on rape show that only a fraction of sexual violence cases are ever prosecuted (BRÅ, 2019a, 2019b, 2020; Lovett & Kelly, 2009, p. 17). A gap such as this, between the number of crimes committed and the number that lead to a criminal justice response such as prosecution or conviction, is often referred to as a “justice gap” (Walby et al., 2011, p. 100). The justice gap for sexual offenses is frequently raised as a problem, and many have argued that the criminal justice system needs to become better at investigating sexual offenses and securing convictions (e.g., Hohl & Stanko, 2015, p. 327).

In this study, we follow police-reported cases of sexual harassment through the criminal justice system by analyzing preliminary investigation case files, prosecutorial decisions, and court judgments. Our overarching question is: What characterizes cases that get filtered out at an early stage of investigation, as compared to cases that get prosecuted? As part of our theoretical framework, we recognize a distinction between formal and substantial criminalization, described below.

## To What Extent Is Sexual Harassment De Facto Criminalized? A Closer Look at Sexual Molestation

### Sexual Molestation

The behaviors we think of in everyday terms as sexual harassment actually fall into one of several different categories of crime under Swedish law, including both sexual offenses (e.g., sexual molestation, sexual coercion, attempted rape) and nonsexual offenses (e.g., molestation, criminal insult, unlawful threat, unlawful persecution, unlawful breach of privacy, intrusive photography). We have chosen to analyze police-reported incidents of the specific offense of sexual molestation (*sexuellt ofredande*) as defined in Chapter 6, Section 10 of the Swedish Criminal Code (Brottsbalken, SFS 1962:700/2022:1043): “A person who … exposes themselves to another person in a manner that is liable to cause discomfort, or who otherwise molests a person by word or deed in a way that is liable to violate that person's sexual integrity is guilty of sexual molestation and is sentenced to a fine or imprisonment for at most two years” (Swedish Government, 2024). The reason for our choice is, first of all, that this offense, as it is defined in law, encompasses various forms of intimate intrusion. Sexual molestation is a catchall provision for criminal acts that cannot be prosecuted as more severe sexual offenses, such as rape or sexual coercion. The aim of the provision is to protect the sexual integrity and sexual self-determination of individuals. The provision on sexual molestation explicitly mentions flashing. Other types of behavior (including physical and verbal intrusions) can also amount to a crime if the behavior violates a person's sexual integrity. The scope of the provision rests on whether the criminal act is of such a nature that, from an objective standpoint, it violates the victim's sexual integrity. This objectified assessment implies that it is not necessary to prove that the conduct had this impact on the victim and, conversely, that the victim's apprehension of the event does not matter. However, the context of the incident needs to be taken into consideration, including the age of the suspect and the complainant, their relationship, and the environment. The scope of the provision is mainly limited by the requirement that the behavior in question be of a sexual character, which excludes, for example, brief touching of a girl's leg (Nytt Juridiskt arkiv avdelning I [NJA], 2018 s. 443) or a woman's thigh (NJA 2018 s. 1091), and isolated verbal comments that include references to breasts (NJA 2024 s. 564) or insults such as “whore” (NJA 1997 s. 359). Up-skirting, however, has been considered sexual molestation (NJA 2017 s. 393), as has repeatedly asking a girl if she wants to sell sex (NJA 2016 s. 129). Typical examples of sexual molestation include groping genitals or breasts, sending pictures of genitals, or sending e-mails and messages that are clearly of a sexual nature. Without going too much into detail about this offense, it is safe to say that the scope of the provision is not always clear, which makes it difficult to predict if any specific incident of sexual harassment will count as a crime or not.

Official crime statistics also show that incidents of sexual molestation are increasingly being reported to the Police Authority. The number of reported instances per 100,000 inhabitants of sexual molestation against persons over 18 years old (flashing excluded) climbed from 18 in 1995 to 58 in 2017 (BRÅ, 2024a). In 2021, the number rose to 61, but fell again in 2023 to 50 reported instances per 100,000 inhabitants.

### De Facto Criminalization

To answer our research question—what characterizes cases that get filtered out at an early stage of investigation, as compared to cases that get prosecuted?—we need to explore to what extent sexual harassment is de facto criminalized. How is the broad statutory definition of sexual molestation applied by such criminal justice actors as police and prosecutors? This aim is theoretically grounded in the complex relationship between law in books and law in action (Davies, 2008). An often-used metaphor presents the legal system as having two faces: law is both a set of norms and a set of legal practices (Tuori, 2002). A characteristic feature of the legal system is the distinction between norm and fact (Luhmann, 2004, p. 72; Tuori, 2002, p. 25–29). The assumption that norms can be described independently of facts generates the understanding of law as a normative system—the legal order. However, norms become meaningful—acquire a precise content—only in relation to facts, that is, when they are applied in a particular case. This distinction between norm and fact explains how law comes to consist of both the legal order and those legal practices that produce and reproduce the legal order. In this study, the legal practices are the decisions made by police officers and prosecutors within the framework of a pretrial investigation. The legal order corresponds to the statutory description of the offense of sexual molestation and its interpretation in authoritative sources of law. The legal practices performed by police investigators and prosecutors produce the norm of sexual molestation, and vice versa.

This conceptualization of law as both legal order and legal practices is mirrored, in the field of criminal law, by the idea that criminalization consists of both formal and substantial criminalization (Lacey, 2009, p. 943). Formal criminalization refers to legislation, while substantial criminalization refers to actual implementation of formal norms. Another way to say this is that in determining the scope of criminalization—to what extent sexual harassment is criminalized—we need to consider adjudication practices, decision-making by prosecutors, the police as agents of criminalization, and the role of ordinary citizens in the criminal justice system (Duff, 2018, pp. 40–49; see also McNamara et al., 2018, p. 93 on “thick” criminalization). We believe that mapping to what extent sexual harassment is substantially, or de facto criminalized by tracking recorded incidents through the criminal justice system can contribute to ongoing discussions about whether criminalization is a suitable response to sexual violence (Douglas et al., 2023; McGlynn, 2024; Wegerstad & Andersson, 2024).

### Research Questions

To understand de facto criminalization, it is necessary to examine the types of situations in which the preliminary investigation is closed at an early stage versus those in which investigative resources are deployed to find a suspect or collect evidence and, eventually, penalties are imposed. We have explored whether there is such a thing as a typical case of criminalized sexual harassment, using a quantitative analysis of case files in police-reported cases of sexual molestation. Our study followed reported cases through the criminal justice system. Attrition studies are often made with the ambition to explore how criminal investigations and the criminal process could be improved or produce more convictions, or with the ambition to explore potential discrimination (BRÅ, 2019b, 2019c; Ferguson, 2016; Jehle, 2012; Nelson, 2012). Our research questions were mainly of a descriptive nature. First, we asked what characteristics were common to the incidents of sexual harassment reported to the Police Authority. By that, we mean mainly where the incidents took place, the relationship between the complainant and suspect, and the nature of the incident itself and its circumstances. This question was related to our aim of describing substantive criminalization, but we also needed this descriptive information to analyze possible differences between cases that were cleared and cases that were closed without further action. Secondly, we wanted to know what happened to incidents of sexual harassment reported to the Police Authority: mainly, this meant what investigative measures were taken and what decisions were made by those in charge of the investigation. Again, we used this descriptive information to analyze possible differences between cases that were cleared and cases that were closed without further action. Finally, we explored differences between cases that were cleared (whether by indictment or the imposition of a summary fine) and cases that were terminated, again with the aim of finding out if there were typical cases of sexual harassment that are de facto criminalized.

### Procedural Aspects

Under Swedish law, the Police Authority or the public prosecutor have a duty rather than the discretion to initiate a preliminary investigation (Wong, 2022, p. 192). The main rule is that a preliminary investigation shall be initiated as soon as there is reason to assume that a crime that is subject to public prosecution has been committed. However, the Code of Judicial Procedure (Rättegångsbalken, SFS 1942:740/2014:649/2004:504) stipulates exceptions where it is obvious that the crime is not possible to investigate (Chapter 23, Section 1, Paragraph 2) or where it can be expected that the investigation, if initiated, will be terminated either because the cost for continued investigation is not in reasonable proportion to what is at stake or due to waiver of prosecution (Chapter 23, Section 4a, Paragraphs 1–2). The first stage of the process leads to one of three final decisions. These are regulated in detail in Chapter 23, Sections 2, 4, 4a, and 22 of the Code of Judicial Procedure, although much scope for discretion remains. The possible final decisions are:

-not to initiate a preliminary investigation (*inte inleda förundersökning*);
-if a preliminary investigation is initiated
    • to close the preliminary investigation with no further action (*förundersökning läggs ned*);
    • to refer the case to the prosecutor for indictment (*förundersökning slutredovisas till åklagare*).

When a case is referred to the prosecutor, the prosecutor decides whether to indict or not. The latter is called a negative decision on prosecution (*negativt åtalsbeslut*). A negative prosecution decision can be made by the prosecutor pursuant to Chapter 21, Section 20 of the Code on Judicial Procedure. They can also impose a summary sanction order (*strafföreläggande*), which means that the prosecutor imposes a fine, or waive a prosecution (*åtalsunderlåtelse*).

The leader of the preliminary investigation, who is responsible for making decisions on an ongoing basis, can be either the Police Authority or a prosecutor. According to the Code of Judicial Procedure (Chapter 23, Section 3, Paragraph 1), a prosecutor is to take over this responsibility from the Police Authority when the matter is not of a simple nature and the police have identified someone who can be reasonably suspected of the crime. The prosecutor is also to take the lead on other cases when special reasons justify it. Alongside the buying of sexual services, sexual molestation is the only sexual offense that is considered to be of a simple nature (ÅFS, 2005:9).

## Method and Material: Quantitative Analysis of 215 Case Files

### Material

Our material consisted of case files retrieved from two police districts in South Sweden: one urban district and one rural. To get the files, we first requested a list of case numbers (*k-nummer*) from the Swedish National Crime Prevention Agency (*Brottsförebyggande rådet*). We requested incidents that were reported and registered as sexual harassment against a woman or man aged 18 or older, and specified incidents that were reported in 2017 or 2018 and where a final decision had been made. We received a list of 255 case numbers. We then requested the case files from the police districts and received 247 files. Of these 247, we excluded 15 cases based on the following exclusion criteria: the file was not complete, the file did not include the crime of sexual harassment, or there was no final decision. We also excluded cases with multiple complainants or multiple suspects, leaving us with 215 cases in total. The study was approved by the Swedish Ethical Review Authority (Reference No. 2019–02953).

The case files consisted—with great variation in scope—of three types of documents: a preliminary investigation report (*förundersökningsprotokoll*), including the initial report (*anmälan*) and, when applicable, interview transcripts, notes on investigative measures, photographs, evidence, and so on; a file history (*ärendehistorik*), which includes dates of measures and decisions taken and the names of involved parties, in chronological order; and a crime report (*brottsrapport*), which includes decisions with reasonings described in brief. Twenty-eight of our cases were referred to the prosecutor. For these cases, we also collected the prosecutor's decision along with the court's written judgment, which was issued in 17 cases that led to indictment, or a summary sanction order, which was issued in three cases. (In the remaining eight cases, the prosecutor made a negative decision on prosecution.)

### Variables

Taking inspiration from an attrition study on rape in Sweden (BRÅ, 2019b), we developed a code scheme with several variables. Three types of data were extracted. The first focused on information about the crime itself and included the *gender* of the complainant and suspect (described as man/woman), *who reported* the incident to the police (the complainant, a friend, or family member, a security guard or police officer, institutional staff, or someone else), whether a *bystander intervened*, and the *time elapsed between the incident and reporting*. Further, data on the *place of incident* and the *relationship between the complainant and the suspect* (see Table 1) were extracted. There were four *place* variables: (a) *private residence*, defined as complainant's home, someone else's home or a care home; (b) *indoors, not private residences*, which included indoors other than home, for example, workplace or hotel; (c) *public setting or outdoors*, including various public environments (e.g., gym, bus, train, shopping mall, indoor pool), public entertainment locale, and outdoor environments such as streets, parks, bus stops, or beaches, as well as taxis and cars; and (d) remote, which was defined as communication via the internet, letter, or telephone. In cases involving multiple incidents, we only coded information for the first incident (5%, or 11 cases, involved more than one incident of sexual harassment). The complainant–suspect relationship included the categories *known* and *stranger*. The “known” category indicates that the suspect was an intimate partner, a friend, or acquaintance, a professional colleague, a family member, or otherwise known to the complainant. The category “stranger” includes suspects whom the complainant encountered in a professional capacity, for example, a customer in a shop where the complainant worked, and someone the complainant had met earlier the same day. Furthermore, the data were coded according to whether the report included *multiple incidents* of sexual molestation, whether *other crimes* were reported at the same time, and whether the suspect had *previously committed crimes* against the complainant. As mentioned earlier, the crime of sexual molestation encompasses a wide range of acts, from physical attacks and exposure to verbal comments, text messages, and the sending of nude or pornographic pictures. We categorized the incidents as one of three *types of sexual molestation*: physical, verbal, and visual. Several forms of molestation could be present in a single case. We coded information about the prevalence of *additional violence*, including mild forms of violence such as pulling the complainant's clothes, and/or threats directed toward the complainant as part of the incident, but not reported as a separate offense. We also coded information in the case files about how the complainant reacted to the suspect's behavior, identifying three *forms of resistance*: verbal, physical, or moving away. Several forms of resistance could be present in a single case.

**Table 1.** Place and Relationship; Sample Size *n* = 215.

	*n*		%	n	%	
*Place of incident*					
Private residence	47	22	Complainant's home	18	8
			Someone else's home	24	11
			Care home	5	2
Indoors, not private residence	26	12	Indoors other than home (e.g., workplace)	24	11
			Hotel	2	1
Public setting or outdoors	101	47	Public environment (e.g., gym, bus, train, shopping mall, indoor pool)	34	16
			Outdoors (e.g., street, park, bus stop, beach)	29	14
			Public entertainment locale (e.g., nightclub, pub)	25	12
			Taxi	11	5
			Car	2	1
Remote	41	19	Internet (including Messenger, Snapchat, WhatsApp)	15	7
			Letter or telephone	26	12
*Complainant–suspect relationship*					
Known	70	33	Intimate relation	20	9
			Friend/acquaintance	19	9
			Professional—colleague	16	7
			Other	10	5
			Family	5	2
Stranger	145	67	Unknown	97	45
			Professional—unknown	45	21
			Met same day	3	1

Next, we extracted data on the investigation. We coded the time elapsed from when the incident was reported until a final decision was made, as shown in Table 2. Information about whether a suspect had been identified relied on two separate definitions. First, we established whether the person reporting the offense had *identified anyone by name* (first and last) to the Police Authority. We then coded whether investigatory measures had been used and whether these measures had led to the identification of a suspect. This information is presented in Table 2. A second definition of “suspect” is the one used by the Police Authority. It relies on a level of assessment of evidence in the case. According to this definition, a suspect is someone who can be *reasonably suspected* (*skäligen* misstänkt; Wong, 2022, p. 190) of committing the crime. In addition, we coded the prevalence of witnesses, as well as whether suspects, complainants, and witnesses were *formally interviewed* by the police. Furthermore, we coded the *leader of the preliminary investigation* (Police Authority/prosecutor), whether the *complaint withdrew* or did not participate to some extent, and whether a suspect was *arrested* (*gripen*) by police officers or security guards, *detained* by order of the prosecutor (*anhållen*) or remanded in *custody* (*häktad*). According to previous studies of the criminal justice system in Sweden, the need for a language interpreter may have a discriminatory effect (BRÅ, 2008). Information about the need for an interpreter among complainants and suspects was coded. This information is recorded on the interview template, meaning it is only available in cases where a formal interview took place.

**Table 2.** Key Characteristics of Police Investigations.

	*n*	%
*Time from incident recorded to final decision; when final decision was made; n* *=* *215*		
Same day	13	6
Day after	46	21
Within a week	37	17
Within a month	38	18
Within 3 months	27	13
Within 6 months	33	15
Within a year	12	6
More than a year	9	4
*A suspect identified, yes or no; n* *=* *215*		
Yes, done by person reporting the incident	101	47
Yes, after measures taken by the police	42	19
No, after measures taken by the police	25	12
No, no measures taken by the police	47	22
*Reasons for terminating a preliminary investigation; n* *=* *165*		
Insufficient evidence, crime cannot be proven	106	64
Investigative efforts have not led to any suspect being linked to the crime	31	19
Complainant backed out	9	5
Not sexual molestation as defined under the law	8	5
Other ground	11	7

**Figure 1.**
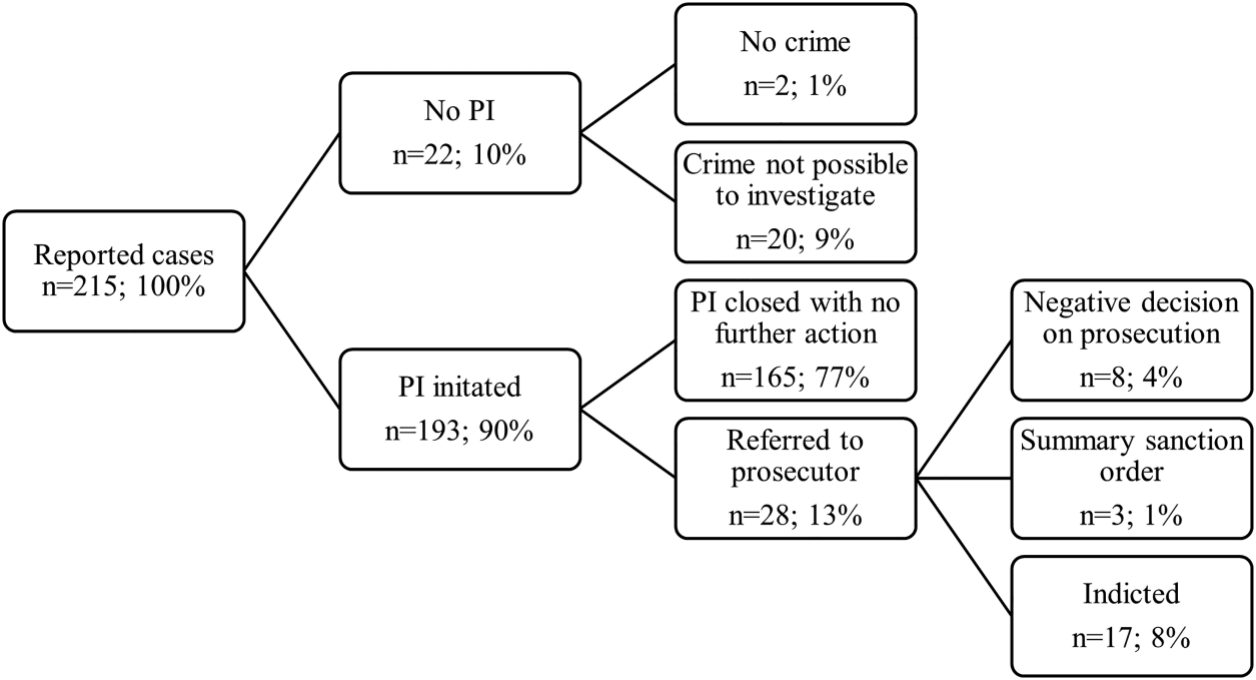
Decisions Made by Police or Prosecutor.

Finally, data was extracted on the type of final decision made by the police. As shown in Figure 1, this decision may be *not to initiate a preliminary investigation*, to *close the preliminary investigation with no further action* or to *refer the case to a prosecutor* for indictment. The decision to close an investigation is often justified using standard phrases. The *reasons for terminating a preliminary investigation* were coded as shown in Table 2. In addition, the *reasons for not initiating a preliminary investigation* were specified into two categories: either no crime had been committed or the crime was not possible to investigate. For the cases referred to the prosecutor, information was collected on the *prosecutor's decision* and coded into three categories: negative decision on prosecution, summary sanction order, and indictment (Figure 1).

### Analysis

It is important to distinguish between attrition rate, which refers to the proportion of cases that “fall out” or are “filtered out” or “dropped” from the criminal justice system, and conviction rate, which refers to the proportion of cases that lead to a conviction (Walby et al., 2011, p. 100). To calculate either one, both a starting point and an end point must be defined. In our material, the starting point is the recording and classification of an incident by the police as a specific crime. As Figure 1 shows, there are several possible “end points.” The decision of whether or not to initiate a preliminary investigation is a first juncture where attrition happens. The next juncture comes with the decision to either close the preliminary investigation with no further action or to refer the case to the prosecutor for possible indictment. A third juncture comes with the prosecutor's decision on how to proceed. If the suspect accepts a summary sanction order, this is equivalent to a conviction in court. The Swedish National Council for Crime Prevention (BRÅ), which is the agency that collects statistics on crime in Sweden, uses the concept of “person-based clearance.” Person-based clearance is a kind of conviction rate, but it is broader and includes when a suspect has been tied to an offense through an indictment, the issuance of a summary sanction order or the issuance of a waiver of prosecution. For comparability with BRÅ's figures, we have defined clearance the same way BRÅ does, which means that “cleared” is defined as either indictment or a summary sanction order; waiver of prosecution did not exist in our material. To calculate the clearance rate, we divided the number of cases resulting in an indictment or summary sanction order by the total number of recorded cases.

We then used cross-tabulation to analyze whether the *clearance rate* (the dependent variable) differed according to the various characteristics of the cases. It was expected that some of the variables would impact the clearance rate. For example, a formal interview with both the complainant and the suspect is required for a case to be referred to the prosecutor. This means that clearance depends on the existence of a formal interview. However, we can also be certain that if no interview took place, there would be no indictment. We chose independent variables that were expected to have little or no impact on the clearance rate. As shown in Table 3, the independent variables were whether the suspect was known to the complainant or a stranger; the place of the incident; whether or not physical harassment occurred; the complainant's reaction (verbal or physical resistance or moving away); whether someone intervened; and whether there were any witnesses. The variable “place of incident” was specified as either a *public place*, consisting of public environment, outdoors, public entertainment locale, and taxi, or *other place than public*. As shown in Table 4, the independent variables related to reporting and investigation were whether the victim or someone else reported the incident, whether the incident was reported on the same day that it occurred, whether the suspect was arrested and whether the complainants and suspects needed an interpreter. We used the Pearson chi-square test to calculate significance levels, except for variables where more than 20% of the cells had expected values of less than five. In such cases, we used Fisher's exact test instead.

**Table 3.** Clearance Rate Depending on Circumstances Regarding the Incident.

Independent variable	Code	Clearance rate %	n cases cleared	*p*-value	Test
Complainant–suspect relationship	Known	4	3	.078	Chi-square
	Stranger	12	17		
Place	Public place*	16	16	.001	Chi-square
	Other place than public*	3	4		
Type of harassment	Physical harassment	12	16	.095	Chi-square
	No physical harassment	5	4		
Complainant reaction	Verbal resistance	12	12	.275	Chi-square
	No verbal resistance	7	8		
	Physical resistance	14	7	.267	Exact test
	No physical resistance	8	13		
	Move away	3	2	.072	Chi-square
	No move away	12	18		
Intervention	Someone intervenes*	25	5	.026	Exact test
	No intervention*	8	15		
Witness	Witness*	21	17	.000	Chi-square
	No witness*	2	3		

*Note.* Clearance rate for all cases: 9%.

*n* = 215.

*p < .05.

**Table 4.** Clearance Rate Depending on Circumstances Regarding the Incident, Reporting, and Investigation.

Independent variable	Code	Clear-ance rate %	*n* cases cleared	*n* valid cases	Clearance rate for valid cases %	*p*-value	Test
Who reports	Victim reports*	6	10	207	10	.003	Exact test
	Person other than victim reports*	22	10				
Time from incident to report	Report same day as incident*	17	17	214	9	.000	Chi-square
	Report later than the day of the incident*	3	3				
Suspect arrested	Suspect arrested*	37	7	142	14	.007	Exact test
	No arrest*	11	13				
Suspect has interpreter	Interpreter	41	9	63	32	.252	Chi-square
	No interpreter	27	11				
Complainant has interpreter	Interpreter	0	0	152	13	.607	Exact test
	No interpreter	14	20				

*Note*. *n* and clearance rate for all cases differs.

*p < .05.

## Results

### Characteristics of Reported Cases and Police Investigations

Sexual molestation reported to the police is highly gendered. In 93% of our cases, the victim was identified or described as a woman (200 cases); in the remaining cases, the victim was identified as a man (7%, 15 cases). We found information about the suspect's gender in 208 cases; in 97% of these (202 cases), the suspect was a man, and in the remaining 3% the suspect was a woman (seven cases).

While in 90% of the cases (193) the complainant was present when the report was made to the police, it was not always the complainant who made the initial contact with the police. In 207 cases, we found information about who reported the incident, and in 79% of those cases, the complainant reported the incident. Others who reported the incident were friend, family, or relative (4%, 9), security guard or police officer (6%, 13), institutional staff (5%, 11), and other (6%, 12). These figures can be set in relation to the fact that in almost one tenth of the cases, a bystander intervened during the incident (9%, 20 cases).

We found information about the time of the incident and the date of reporting in 214 cases. Almost half of the incidents were reported the same day that the incident took place (48%, 102). 90% of the incidents were reported within a month (191), and 99% were reported within a year (200).

Table 1 shows where the incidents took place. A rather large share of the incidents took place in public settings or outdoors: almost half of the cases (101 or 47%). In about one-fifth of the cases, the place of incident was a private residence (47 or 22%). Table 1 further shows that in one-third of the sexual harassment cases in total, the offender was someone the complainant knew, whereas in two-thirds of cases, the suspect was a stranger to the complainant. In 21% of cases (45), the suspect was a stranger, but a relation of a professional character existed (the suspect was a taxi driver, a security guard, or staff worker; in one case, the complainant was a saleswoman and the suspect a customer), which could potentially make it easier to identify a suspect as compared with a “total stranger.” For cases in the “known” category, the place of the incident was very seldom a public setting (five cases or 7%).

Almost all the cases (204 or 95%) included only one incident recorded as sexual molestation. However, in almost one-fourth of the cases (51 or 24%), other crimes were reported at the same time. In cases where multiple crimes were reported, there was no large difference regarding suspect–complainant relationship: the suspect was known to the complainant in 30 cases (59%) and unknown in 21 cases (41%). The complainant reported that the suspect had previously committed crimes against him/her, that is, repeated harassment, in 38 cases (18%). Among those, the suspect was known to the complainant in 28 cases (74%).

The incidents that are reported and registered as sexual molestation largely involve physical assault. Fourteen percent of cases (29) involved more than one type of sexual molestation. Incidents involving physical molestation alone made up 49% of cases (106); incidents involving verbal molestation alone made up 20% (43), and incidents involving visual molestation alone made up 17% (37 cases).

Further, we found that in one-fifth of the cases (20% or 42 cases), additional violence, including mild forms of violence such as pulling the complainant's clothes, and/or threats, had been directed toward the complainant as part of the incident. The complainant reacted to the suspect's behavior with verbal resistance (48% or 104 cases), physical resistance (24% or 51 cases), and/or walked or moved away (27% or 58 cases).

We now turn to describing what kinds of decisions and measures are taken by the police when they record an incident. As Table 2 shows, sexual molestation cases are handled rather rapidly. Within a week, 44% of our cases had reached a final decision. This indicates that there are not that many investigatory measures taken. Within 6 months, 90% of all the cases had been either closed or referred to the prosecutor.

Table 2 further shows whether and how a suspect was identified. In two-thirds of the cases, a suspect was identified by name. In 92 cases or 43%, the identified suspect was considered a “reasonable suspect.” Our material shows that even when there was a “reasonable suspect,” this did not mean that the suspect was interviewed. Of the 92 cases where a reasonable suspect was identified, such suspects were formally interviewed in 62 of them; in other words, one-third of such suspects were not interviewed. Of the 62 cases where an interview with the suspect was conducted, most suspects denied culpability (86%, 53 cases). In five cases (8%), the suspect confessed, whereas in four cases (6%) the suspects’ position regarding the charge was unclear (6%). Neither was the complainant always formally interviewed. Almost one-third of the complainants (64 in all, or 30%) were never interviewed apart from being asked for details when the incident was first reported. There was an identified witness in 83 of the total cases (39%). In 16 of these cases (19%), no witness was interviewed.

The Police Authority, not a prosecutor, regularly heads preliminary investigations in sexual molestations: in 85% (183) of our cases, the Police Authority was in charge of the investigation.

We found information about complainant withdrawal or lack of participation in 11% (23) of the 215 cases.

Regarding investigative measures, a suspect was arrested (*gripen*) by police officers or security guards in 19 cases (9%) and subsequently detained by order of the prosecutor (*anhållen*) in three cases (1%). In no case was a suspect remanded in custody (*häktad*).

Of the 152 cases where the complainant was interviewed, the complainant needed an interpreter in nine cases (6%). Of the 63 cases where the suspect was interviewed, the suspect needed an interpreter in 22 cases (35%).

As Figure 1 shows, a preliminary investigation was initiated in 193 cases (90%). Of these, 165 were terminated with no further action. As Table 2 shows, the most common reason for terminating a preliminary investigation is that the crime cannot be proven due to insufficient evidence. The second most common reason is that no suspect can be linked to the crime.

### An Overall Clearance Rate of 9%, but the Clearance Rate Differs

Figure 1 shows those final decisions taken by the Police Authority or a prosecutor, which means that a case will be discontinued. In our material, 22 out of 215 cases (10%) ended with the decision not to initiate a preliminary investigation. Of the 28 cases referred to the prosecutor, 17 cases proceeded to indictment and three ended with the issuing of a summary sanction order (*strafföreläggande*). The remaining eight of 28 cases were closed by the prosecutor; that is, the prosecutor made a negative decision on prosecution (*negativt åtalsbeslut*). The reason in all eight cases was that there were insufficient grounds to prosecute, as a conviction could not be expected. Of the 17 cases that proceeded to indictment (and trial), all led to a conviction in the court of first instance (the District Court). Just one of those convictions was later overturned by the Court of Appeal. This means that, basically, in our material, a decision to prosecute equals a conviction.

The clearance rate—the number of cases with indictment or summary sanction order divided by all recorded cases—was 9%.

As Table 3 shows, the clearance rate was higher for cases where the suspect was a stranger to the victim, for cases where the incident occurred in a public place, and for cases where the incident included some kind of physical sexual harassment. The clearance rate was also higher in cases where the complainant offered some kind of verbal or physical resistance (saying no, shoving the suspect, etc.). However, if the complainant reacted by leaving the crime scene or moving away from the suspect, the clearance rate was lower. Among those factors, only place was statistically significant. Our analysis further showed that if someone intervened and if there were witnesses, the clearance rate was higher, and both were statistically significant.

We should note that we added the column “Clearance rate for *valid* cases” to Table 4. That is because not all 215 cases had the relevant information. The number of valid cases refers to cases with the relevant information. For example, we only had information about who made the report in 207 cases. Table 4 shows that the clearance rate was higher in cases where a person other than the victim reported the incident. Table 4 further shows that the clearance rate was higher if the incident was reported on the same day as it occurred. If the suspect was arrested, the clearance rate was higher. We calculated the clearance rate based on the number of cases where there was an identified suspect, which was 142 cases.

Finally, regarding whether the need for an interpreter could impact the clearance rate. We calculated the clearance rate based on cases where a formal interview had been conducted. We obtained no significant results, but we did find a difference regarding whether it was the suspect or the complainant who needed an interpreter (Table 4). The clearance rate was higher, 41%, when the suspect needed an interpreter than when they did not, 27%, whereas the opposite was true for the complainant. In cases where the complainant needed an interpreter, the clearance rate was 0%.

## Discussion

### Real Sexual Harassment?

We looked for possible differences between cases that are filtered out at an early stage of criminal investigation and cases that are prosecuted. We found differences in clearance rate that indicate that physical attacks by strangers in public places are more likely than other kinds of sexual harassment to be prosecuted. One explanation for these results might be that the prospects for investigation increase if the incident occurs in a public place. If the incident takes place in a public setting, there are more likely to be people there who intervene and report the incident to the Police Authority immediately. There are more likely to be witnesses to the incident. The two factors that someone intervenes and the existence of witnesses are probably measuring the same thing to some extent—the person who intervenes is also a witness. The fact of someone intervening should also be considered in relation to who reports the incident, as shown in Table 4. We believe that if someone intervenes, that person might also be the one who makes the report. The existence of witnesses, the presence of someone who intervenes, and someone else than the complainant reporting to the Police Authority are all variables that should be seen as related to the higher clearance rate for incidents occurring in public places. And if security guards or the police immediately respond to the report, it is more likely that a suspect will be arrested on the spot. We believe that the two variables, time to report and arrested suspect, are interrelated, because if an incident is reported the same day it happens, the possibility of an arrest is greater. In this respect, our findings confirm results from earlier studies that show the importance of making an arrest for later bringing charges and eventually producing a conviction (BRÅ, 2019c; Nelson, 2012).

Another interpretation of our findings is that physical attacks by strangers in public places correspond to the concept of “real rape” that has been developed in research about police-reported rapes and how they are processed by the criminal justice system (coined by Estrich, 1987; Hohl and Stanko, 2015). “Real rape” has been used to denote incidents of rape that could typically be characterized as an unknown man attacking a woman in a public place (e.g., Andersson et al., 2019). From this perspective, attacks by strangers are seen as “real sexual harassment” and are taken more seriously than other, “messier” kinds of sexual harassment incidents. A stranger is closer to the ideal perpetrator than a work colleague or a partner (Christie, 1986). The finding that a suspect's need for an interpreter raises the clearance rate, while a complainant's need for an interpreter lowers the clearance rate, suggests that police investigations are influenced by perceptions that real victims are native Swedish speakers and real perpetrators are nonnative speakers (BRÅ, 2008). Due to the small number of cases in our study, this finding needs to be further researched, but it does gain support from a study by Lainpelto and Bisso (2019), which shows that in Swedish media discourse, public sexual harassment and sexual violence have been framed as an “immigration problem.” The study found that this discourse has had an impact on prosecution and court practice in cases of sexual molestation: young people with foreign backgrounds were less frequently acquitted and were also found guilty in cases with less robust evidence, compared to suspects and defendants with a Swedish background.

It could also be that formal criminalization, despite the inclusive wording of the statute, still privileges physical assault as the typical case of sexual molestation. In addition, some uncertainty might reign about what counts as sexual molestation, specifically regarding the taking and sending of pictures. As mentioned, in recent years, the Supreme Court has adjudicated several cases having to do with the boundaries of sexual molestation, which could signal that such uncertainty exists. In our material, however, only 5% of terminated cases were closed for the stated reason that the incident was not sexual molestation as defined by law (Table 2).

### Is More Substantial Criminalization the Answer?

The aim of this study was to explore to what extent sexual harassment is de facto criminalized. Our study shows that nine out of ten incidents of sexual harassment reported to the Police Authority do not lead to a perpetrator being held accountable. At first glance, this figure suggests very little substantial criminalization of sexual harassment, as well as a lack of efficiency in the criminal justice system. If that is true, this lack of efficiency is not specific to the crime of sexual molestation. In comparison with other crimes, 9% clearance rate is not an exceptional figure. According to official crime statistics, the clearance rate for all crimes varied between 12% and 15% in Sweden during the years 2014–2023. For crimes against a person, the figure varied between 8% and 11% (BRÅ, 2024b). For assaults (including aggravated assault), the clearance rate reported by BRÅ was between 11% and 13% during the years 2014–2023.

Our finding that slightly more than one-fourth of the cases in our study were terminated the same day they were reported or the day after, indicates that there was room for more investigative measures to be taken. That said, there might be good reasons for terminating a case early. Some of our findings, however, indicate that more could be done in individual cases. For example, we found that almost one-third of the complainants were never interviewed apart from being asked to provide details when the incident was initially reported. Neither were all witnesses nor suspects interviewed. For comparison, it has been recommended, as a measure to improve the clearance rate in rape cases, that anyone identified as a “reasonable suspect” should be interviewed (BRÅ 2019b). The fact that the law designates sexual molestation as a less severe offense than, for example, rape, could then matter for substantial criminalization (it is, in part, this legal designation that empowers the Police Authority, as opposed to a prosecutor, to head the preliminary investigation).

Another perspective on the clearance rate is what the expectations of reporting a case to the police are. In Sweden, low clearance and conviction rates became one argument (among others) for modifying the legal definition of rape (Dir. 2014:123), but such a debate concerning sexual harassment has not (yet) been initiated. Perhaps, compared to rape, the expectations that the criminal justice system holds a perpetrator accountable when it comes to sexual harassment are lower. Some support for that argument is provided by Mellgren et al. (2017), who asked why respondents did not report sexual harassment. One theme in their answers was that “the police would not do anything anyway,” indicating a mistrust of the criminal justice system. The reason for reporting sexual harassment is not necessarily always to get a conviction. In an interview study based on self-reported exposure to sexual offenses registered by the Swedish National Council for Crime Prevention (BRÅ, 2019a, p. 78), one theme that emerged concerning reporting to the police was the importance for victims of marking that the behavior was unwanted. That could involve telling a security guard what had happened. Further, some respondents said that they considered reporting everything that happened to the police to make it clear how common it is for women to be subjected to sexual violence.

The rather low clearance rate in our study can also be understood as evidence that the criminal justice system is not the right solution for preventing sexual harassment and providing justice to victims. Other kinds of interventions are necessary. Fileborn and Vera-Gray argue that the lack of criminal justice responses to street harassment “provides the opportunity to develop justice responses to street harassment from the starting point of victim's justice interests” (2017, p. 204). They show that for the respondents in their study, the most important conceptualization of justice was transformative justice—education, prevention, and transforming gender norms. Mellgren et al. (2017) suggest the use of bystander programs and address the role of security staff at nightclubs, who can contribute to, or counteract, a culture of masculinity and sexism at these venues. Feminist self-defense has also been suggested as a way forward (Vera-Gray & Kelly, 2020).

### Methods for Measuring Attrition

Lastly, we want to draw attention to methods for measuring attrition. We found a clearance rate for all cases of 9%. An alternative way to calculate the clearance rate could be to include only cases where a preliminary investigation was initiated (193 cases). This would yield a clearance rate of 10.3%. In most of the cases where a preliminary investigation was *not* initiated (20 of 22 cases), the stated reason was that it was obvious that the crime was not possible to investigate. In just two cases, the stated reason was that there were no grounds to assume that an offense subject to public prosecution had been committed. This means that in 213 out of 2015 cases, there was reason to assume that an offense subject to public prosecution had been committed. The clearance rate does not change much whether the total amount of reported cases is 213 (9.4%) or 215 (9.3%).

A clearance rate for all cases of 9% is a bit lower than the numbers provided by official crime statistics (BRÅ, 2024b). For the period we looked at, 2017–2018, the official clearance rate for sexual molestation against adults (flashing excluded) was 12% in 2017 and 14% in 2018. In 2019–2023, the clearance rate varied between 13% and 17%. The clearance rate for the specific kind of sexual molestation known as flashing is reported separately in BRÅ statistics and lumps together cases of flashing against both minors and adults. The clearance rate for flashing was 14% and 12% for the years 2017 and 2018, respectively, but in subsequent years it varied widely, from 21% to 48%. However, flashing accounts for only a small amount of all reported sexual molestation cases.

For comparability with BRÅ's figures, we have defined clearance the same way BRÅ does. That said, a straightforward comparison is not really possible, because BRÅ does not track cases through the system as we have done. Instead, they define clearance rate as “the number of offences with person-based clearances during one year as a percentage of the number of processed offences during the same year” (BRÅ, 2024c). This method has been criticized for not accurately describing the real proportion of cleared cases (Diesen & Diesen, 2013, pp. 26–31). Tracking recorded cases through the criminal justice system gives a more accurate clearance rate. Our study indicates that the real clearance rate is lower than official crime statistics suggest.

### Limitations and Suggestions for Further Research

A limitation of the study is its relatively small sample size, comprising only two police districts in Sweden, and the small number of cleared cases. Further research is necessary to investigate the differences between cleared and terminated cases. In particular, the impact on the clearance rate of the need for language assistance suggests that it would be worthwhile to investigate how this need affects the criminal justice system. The differences in clearance rates suggest a possible correlation between the examined factors and the clearance rate. However, we cannot rule out the impact of other unconsidered factors (confounders) or the interrelationships within the set of variables included in our analyses. More advanced statistical analysis is recommended.

One challenge when analyzing police files is the lack of consistency in the material. For instance, some case files contained detailed incident descriptions, while others provided information about the incident in more broad terms. Furthermore, information about the suspect was only available where there was actually a suspect, so we had to decide when a person should be considered a suspect. Thus, our coding involved a relatively large margin for interpretation. We suggest that qualitative research of preliminary investigations could provide more insight.

While sexual molestation can be committed against both children and adults, our study includes only complainants over age 18. Young women are particularly vulnerable to sexual harassment (BRÅ 2019a). Future research should examine how cases involving minors that are reported to the police are handled within the justice system.

A final limitation of our study is that we did not explore why investigative measures are taken in some cases but not in others. One question for future research is whether further investigative measures could lead to the identification of suspects in more cases. Another question is what impact it would have on the clearance rate if a prosecutor oversaw the preliminary investigation, as opposed to the current situation, where the vast majority of sexual molestation investigations are led by the Police Authority.
